# Crystal structures of (*N*-methyl-*N-*phenyl­amino)(*N*-methyl-*N*-phenyl­carbamoyl)sulfide and the corresponding disulfane

**DOI:** 10.1107/S2056989015018289

**Published:** 2015-10-24

**Authors:** Matthew J. Henley, Alayne L. Schroll, Victor G. Young, George Barany

**Affiliations:** a Department of Chemistry, University of Minnesota, Minneapolis, MN 55455, USA; b Department of Chemistry, Saint Michael’s College, Colchester, VT 05439, USA

**Keywords:** crystal structure, organosulfur chemistry, sulfide, disulfane, hydrogen bonding

## Abstract

The title compounds, (*N*-methyl-*N-*phenyl­amino)(*N*-methyl-*N*-phenyl­car­bam­oyl)sulfide, C_15_H_16_N_2_OS, (I), and (*N*-methyl-*N*-phenyl­amino)­(*N*-methyl-*N*-phenyl­carbamo­yl)disulfane, C_15_H_16_N_2_OS_2_, (II), are stable derivatives of (chloro­carbon­yl)sulfenyl chloride and (chloro­carbon­yl)disulfanyl chloride, respectively. The torsion angle about the S—S bond in (II) is −92.62 (6)°, which is close to the theoretical value of 90°. In the crystal of (II), non-classical inter­molecular C—H⋯O hydrogen bonds form centrosymmetric cyclic dimers [graph set *R*_2_^2^(10)], while inter-dimer C—H⋯S inter­actions generate chains extending along the *b* axis.

## Abstract 2

(*N*-Methyl-*N*-phenyl­carbamoyl)(*N*-methyl-*N*-phenyl­amino)sulfide and the corresponding disulfane are stable derivatives of (chloro­carbon­yl)sulfenyl chloride and (chloro­carbon­yl)disulfanyl chloride, respectively.

## Chemical context   

As part of a multifaceted program in synthetic and mechanistic organosulfur chemistry (Barany *et al.*, 1983 ▸; Barany & Mott, 1984 ▸; Schroll & Barany, 1986 ▸; Schrader *et al.*, 2011 ▸, and references cited therein), we frequently encounter challenging-to-characterize compounds with one or more reactive acid chloride and/or sulfenyl chloride moieties. These are converted to the corresponding stable carbamoyl and/or sulfenamide derivatives, which are often crystalline, through their reliable, rapid, and high-yield reactions with *N*-methyl­aniline.
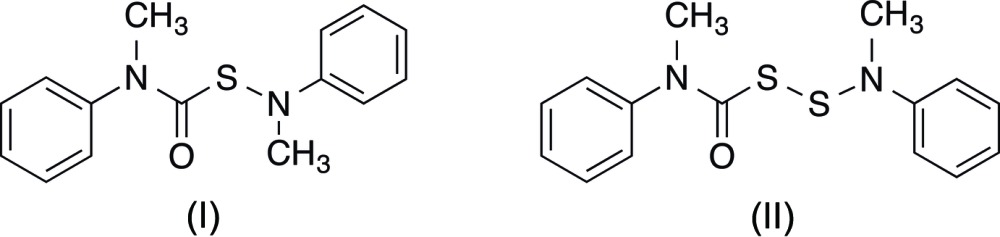


The present paper reports the structures of two such derivatives, *i.e.* (*N*-methyl-*N-*phenyl­amino)(*N*-methyl-*N*-phenyl­car­bamoyl)sulfide (I) and (*N*-methyl-*N*-phenyl­amino)(*N*-methyl-*N*-phenyl­carbamo­yl)­disulfane (II), as determined by X-ray crystallography. The title compounds are derived respectively from (chloro­carbon­yl)sulfenyl chloride and (chloro­carbon­yl)disulfanyl chloride, which are noxious, distillable liquids. They are the first two members of a general family of compounds with the structure Ph(Me)N(C=O)S_*n*_N(Me)Ph, in which the higher members (*n* = 3–6) were found, but not isolated in crystalline form, as components in the reactions of *in situ* generated (2-propoxydi­chloro­meth­yl)(chloro­carbon­yl)polysulfanes with *N*-methyl­aniline (Schroll & Barany, 1986 ▸).

## Structural commentary   

**Figure 1:**
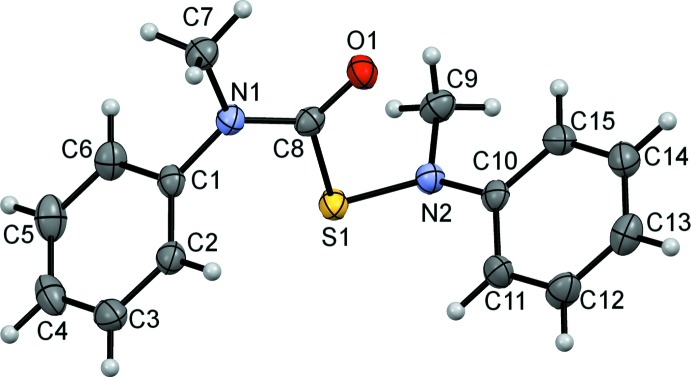
The mol­ecular conformation of compound (I), showing 50% probability displacement ellipsoids with all non-H atoms labeled and numbered.

**Figure 2:**
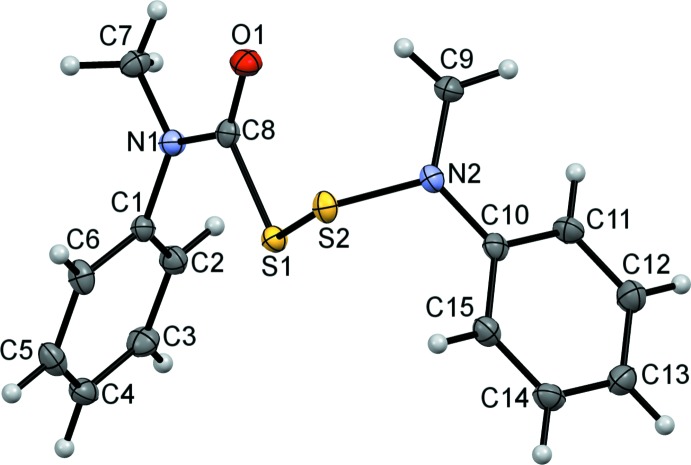
The mol­ecular conformation of compound (II), showing 50% probability displacement ellipsoids with all non-hydrogen atoms labeled and numbered.

**Figure 3:**
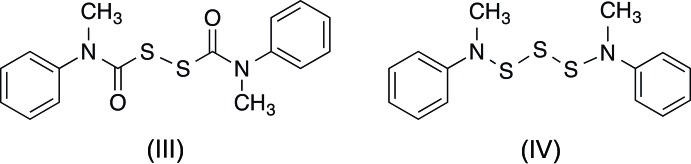
Structures of selected comparison compounds, bis­(*N*-methyl-*N*-phenyl­carbamo­yl)disulfane, (III), and bis­(*N*-methyl-*N*-phenyl­amino)­tris­ulfane, (IV)

The title compounds differ by the number of sulfur atoms: one in (I) (Fig. 1 ▸) *versus* two in (II) (Fig. 2 ▸), and by the resulting relative orientations of the Ph(Me)N(C=O)S and N(Me)Ph moieties. Otherwise, they share similar bond lengths and angles across all analogous bonds (Table 1 ▸). Furthermore, the mol­ecular parameters are all within expected ranges. The S—S bond of (II) is 2.0625 (5) Å, which is comparable to the bond length in elemental sulfur, S_8_ (2.07 Å), but slightly longer than the 2.03 Å found for bis­(*N*-methyl-*N*-phenyl­carbamo­yl)di­sul­fane ([Ph(Me)N(C=O)S]_2_) (III) (Schroll *et al.*, 2012 ▸). In compound (III) (Fig. 3 ▸), the slight shortening of the S—S bond was attributed to a partial double-bond character imparted by the adjacent carbonyl groups. Because (II) is essentially (III) minus one carbonyl group, it is not surprising for the S—S bond length in (II) to be closer to that in S_8_. The torsion angle about the S—S bond in (II) is −92.62 (6)°, which is comparable to the theoretical optimum of 90° (Pauling, 1949 ▸; Torrico-Vallejos *et al.*, 2010 ▸).

## Supra­molecular features   

**Figure 4:**
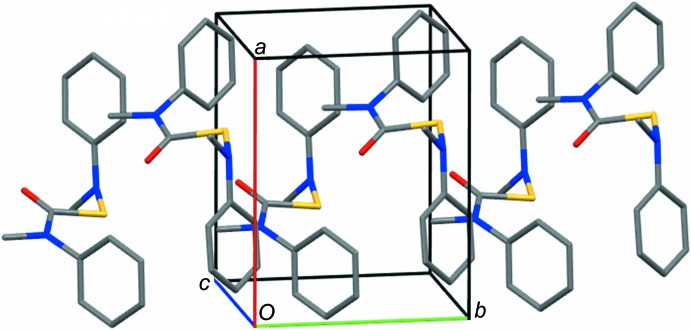
Crystal packing of (I). H atoms are not shown.

**Figure 5:**
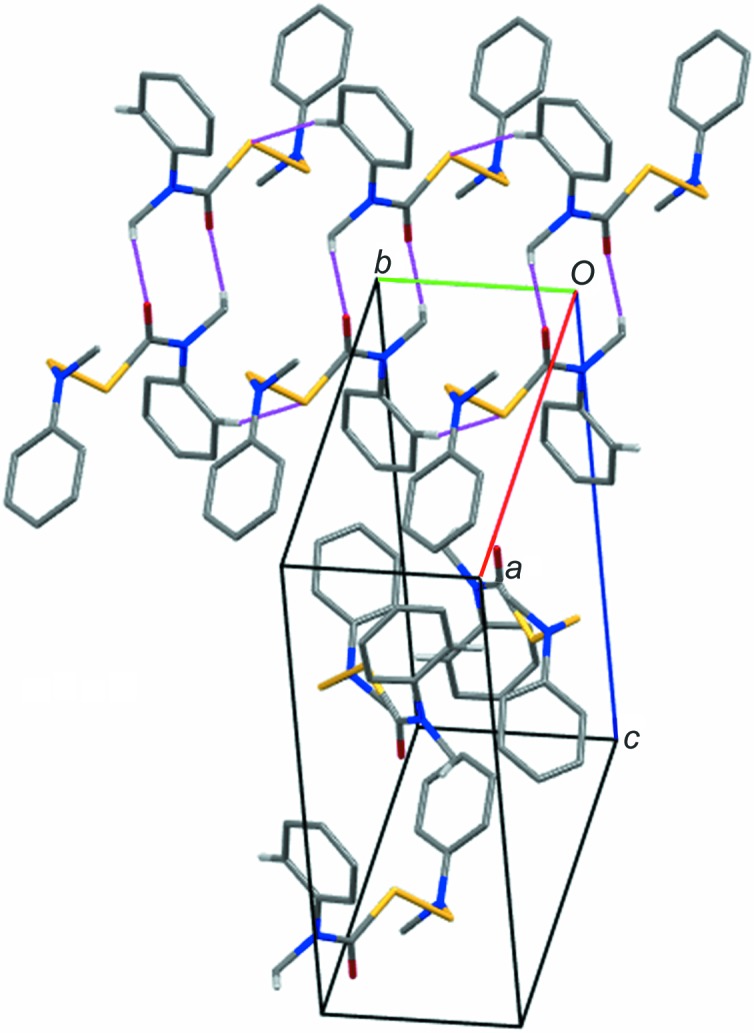
Crystal packing of (II). Only H atoms involved in inter­molecular C2—H⋯S1 and C7—H⋯O1=C8 non-classical hydrogen bonds are shown.

The unit cell of (I) contains two mol­ecules related by a twofold screw axis (Fig. 4 ▸). There are no inter­molecular contacts in the crystal structure of (I). In the crystal of (II) non-classical inter­molecular C7—H⋯O1 hydrogen bonds (Table 2 ▸) form centrosymmetric cyclic dimers [graph set 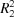
(10)]. Chains of mol­ecules extending along the *b* axis result from inter-dimer C2—H⋯S1 inter­actions (Fig. 5 ▸).

## Database survey   

A search for similar structures in the Cambridge Structural Database (CSD; Version 5.36, update of November 2014; Groom & Allen, 2014 ▸) gave bis­(*N*-methyl-*N*-phenyl­car­bam­o­yl)di­sulfane (III), published previously from our research (Schroll *et al.*, 2012 ▸), as well as two similar bis(car­bamoyl)disulfanes (Bereman *et al.*, 1983 ▸; Li *et al.*, 2006 ▸). Structures containing a similar sulfenamide moiety were absent from the CSD, although two structures reported N—S bonds connected to ‘imido’ [(*R*C=O)_2_N] moieties (Farrell *et al.*, 2002 ▸; Ul-Haque & Behforouz, 1976 ▸). A very recent report from our research describes bis­(*N*-methyl-*N*-phenyl­amino)­tris­ulfane (IV) (Fig. 3 ▸) (Barany *et al.*, 2015 ▸), an *N*-methyl­anilide which contains two ‘sulfenamide’ ends [whereas (III) contains two ‘carbamo­yl’ ends]. Not surprisingly, many geometric parameters of (III) and (IV) superimpose onto the corresponding portions of (I) and (II). For example, the sulfenamide N2—S bond lengths of (I) [1.6784 (15) Å] and (II) [1.6660 (11) Å] are close to that of (IV) [average N—S bond length of 1.657 Å] and the carbamoyl N1—C8 and S1—C8 bond lengths of (I) [1.351 (3) and 1.824 (2) Å, respectively] and (II) [1.357 (2) and 1.827 (1) Å, respectively] are similar to that of (III) [1.345 (3) and 1.825 (2) Å, respectively]. In addition, the torsion angles about the N1—C8 bond of (I) [3.3 (2)°] and (II) [9.16 (15)°] are similar to that of (III) [−6.4 (3)°] and the torsion angle about the N2—S bond in (I) [77.3 (2)°] and (II) [−72.86 (10)] are similar but slightly smaller than that of (IV) (average angle 80.3°).

## Synthesis and crystallization   

The title compound (I) was prepared on scales of up to 0.1 mol by addition of a 0.5 *M* solution of (chloro­carbon­yl)sulfenyl chloride in CHCl_3_ to an equal volume of a 2 *M* solution of *N*-methyl­aniline in CHCl_3_ at 273 K, followed by stirring for 30 min at 298 K (Barany *et al.*, 1983 ▸). Workup by washing with equal volumes of 1 *N* aqueous HCl (3×) and brine (once), drying (MgSO_4_), filtering, and concentrating *in vacuo* gave the product as an oil (nominally qu­anti­tative), and recrystallization from hot hexa­nes (30 mL g^−1^) gave a white solid (typically 65–80% recovery), m.p. 338–340 K, which was stable for several decades when stored under ambient conditions. ^1^H NMR (300 MHz; CDCl_3_): δ 7.43–7.48 (*m*, 3H), 7.37 (*dd*, *J* = 1.9, 7.9 Hz, 2H), 7.23–7.29 (*m*, 2H), 7.12 (*dd*, *J* = 1.0, 8.8 Hz, 2H), 6.86 (*t*, *J* = 7.2 Hz, 1H), 3.41 (*s*, 3H), 3.31 (*s*, 3H). X-ray quality crystals were obtained by dissolving (I) (100 mg) in minimal CHCl_3_ (200 µL) and then adding hexane (2 mL), followed by slow evaporation of the solvent at 298 K over two days.

To prepare compound (II), a solution of (chloro­carbon­yl)disulfanyl chloride (Schroll & Barany, 1986 ▸) (814 mg, 5.0 mmol) in CH_2_Cl_2_ (15 mL) was added over 10 min to a stirred solution of *N*-methyl­aniline (2.2 mL, 20 mmol) in CH_2_Cl_2_ (11 mL) at 273 K. The homogeneous reaction mixture was allowed to warm to 298 K, stirred an additional 30 min, and standard extractive workup [compare to procedure above for (I)] gave the product as a brown oil (1.44 g, 94% crude yield). The crude product was purified by flash column chromatography, eluting with hexa­ne–ethyl acetate (8:1), to provide a yellow oil (1.37 g), which after storing under hexa­nes at 253 K overnight produced the title product as an off-white solid (757 mg, 2.5 mmol, 50%), m.p. 326–327 K (lit. 325–327 K; Barany & Mott, 1984 ▸). ^1^H NMR (300 MHz; CDCl_3_): δ 7.36–7.41 (*m*, 3H), 7.2–7.3 (*m*, 6H), 6.9–7.0 (*m*, 1H), 3.40 (*s*, 3H), 3.37 (*s*, 3H). X-ray quality crystals were prepared by dissolving (II) (23 mg) in CH_2_Cl_2_ (100 µL) and then adding heptane (200 µL), followed by slow evaporation of the solvent at 278 K over 11 days.

## Refinement   

Crystal data, data collection and structure refinement details are summarized in Table 3 ▸. Hydrogen atoms were included at calculated positions [C—H(aromatic) = 0.95 Å or C—H(meth­yl) = 0.98 Å] and treated as riding, with *U*_iso_H = 1.2*U*_eq_C(aromatic) or 1.5*U*_eq_C(meth­yl). With (I), although of no importance in this achiral mol­ecule, the Flack absolute structure factor (Parsons *et al.*, 2013 ▸) was determined as 0.05 (3) for 1450 Friedel pairs.

## Supplementary Material

Crystal structure: contains datablock(s) I, II, 1. DOI: 10.1107/S2056989015018289/zs2342sup1.cif

Structure factors: contains datablock(s) I. DOI: 10.1107/S2056989015018289/zs2342Isup4.hkl

Structure factors: contains datablock(s) II. DOI: 10.1107/S2056989015018289/zs2342IIsup5.hkl

Supporting information file. DOI: 10.1107/S2056989015018289/zs2342Isup4.cml

Supporting information file. DOI: 10.1107/S2056989015018289/zs2342IIsup5.cml

CCDC references: 1428652, 1428651

Additional supporting information:  crystallographic information; 3D view; checkCIF report

## Figures and Tables

**Table 1:** Selected geometric parameters for compounds (I) and (II) (Å, °) Note that when S is not numbered, it is S1 for compound (I) and S2 for compound (II). To specify certain torsion angles, the last atom in the linear structure differs between the two compounds, so *X* is used in place of an atom label.

	(I)	(II)
N1—C8	1.351 (3)	1.357 (2)
S—N2	1.678 (2)	1.666 (1)
S1—C8	1.824 (2)	1.827 (1)
S1—S2	–	2.0625 (5)
		
C9—N2—S	115.90 (14)	116.23 (8)
C10—N2—S	118.74 (12)	118.86 (8)
C10—N2—C9	118.37 (17)	118.17 (11)
		
C1—N1—C8—S1	3.3 (2)	9.16 (15)
N1—C8—S1—*X*	172.19 (14)	−165.53 (8)
C8—S1—S2—N2	–	−92.62 (6)
C10—N2—S—*X*	77.3 (2)	−72.86 (10)

**Table 2:** Hydrogen-bond geometry (Å, °) for (II)

*D*—H⋯*A*	*D*—H	H⋯*A*	*D*⋯*A*	*D*—H⋯*A*
C2—H2*A*⋯S1^i^	0.95	2.84	3.766 (1)	165
C7—H7*B*⋯O1^ii^	0.98	2.60	3.532 (2)	160

Symmetry codes: (i) 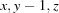; (ii) 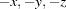.

**Table 3:** Experimental details

	(I)	(II)
Crystal data		
Chemical formula	C_15_H_16_N_2_OS	C_15_H_16_N_2_OS_2_
*M*_r_	272.36	304.42
Crystal system, space group	Monoclinic, *P*2_1_	Monoclinic, *P*2_1_/*c*
Temperature (K)	173	123
*a*, *b*, *c* (Å)	9.0682 (7), 6.8402 (5), 11.4686 (9)	16.0414 (17), 5.5023 (6), 17.2986 (19)
β (°)	103.349 (1)	105.564 (1)
*V* (Å^3^)	692.16 (9)	1470.9 (3)
*Z*	2	4
Radiation type	Mo *K*α	Mo *K*α
μ (mm^−1^)	0.23	0.36
Crystal size (mm)	0.40 × 0.35 × 0.12	0.41 × 0.18 × 0.12

Data collection		
Diffractometer	Bruker SMART APEXII	Bruker APEXII CCD
Absorption correction	Multi-scan (*SADABS*; Bruker, 2002 ▸)	Multi-scan (*SADABS*; Bruker, 2002 ▸)
*T*_min_, *T*_max_	0.687, 0.746	0.699, 0.746
No. of measured, independent and observed [*I* > 2σ(*I*)] reflections	8061, 3145, 2961	16044, 3355, 3033
*R*_int_	0.022	0.024
(sin θ/λ)_max_ (Å^−1^)	0.648	0.649

Refinement		
*R*[*F* ^2^ > 2σ(*F* ^2^)], *wR*(*F* ^2^), *S*	0.027, 0.067, 1.05	0.028, 0.070, 1.06
No. of reflections	3145	3355
No. of parameters	174	183
No. of restraints	1	0
H-atom treatment	H-atom parameters constrained	H-atom parameters constrained
Δρ_max_, Δρ_min_ (e Å^−3^)	0.19, −0.15	0.32, −0.22
Absolute structure	Flack *x* determined using 1285 quotients [(*I* ^+^)−(*I* ^−^)]/[(*I* ^+^)+(*I* ^−^)] (Parsons *et al.*, 2013 ▸)	–
Absolute structure parameter	0.05 (3)	–

Computer programs: *APEX2* and *SAINT* (Bruker, 2002 ▸), *SHELXS97* and *SHELXTL* (Sheldrick, 2008 ▸), *SHELXL2014* (Sheldrick, 2015 ▸) and *Mercury* (Macrae *et al.*, 2008 ▸).

## supplementary crystallographic information

### (I) (N-Methyl-N-phenylamino)(N-methyl-N-phenylcarbamoyl)sulfide Crystal data

**Table:**

C_15_H_16_N_2_OS	*F*(000) = 288
*M**_r_* = 272.36	*D*_x_ = 1.307 Mg m^−^^3^
Monoclinic, *P*2_1_	Mo *K*α radiation, λ = 0.71073 Å
*a* = 9.0682 (7) Å	Cell parameters from 2915 reflections
*b* = 6.8402 (5) Å	θ = 2.3–27.4°
*c* = 11.4686 (9) Å	µ = 0.23 mm^−^^1^
β = 103.349 (1)°	*T* = 173 K
*V* = 692.16 (9) Å^3^	Plate, colourless
*Z* = 2	0.40 × 0.35 × 0.12 mm

### (I) (N-Methyl-N-phenylamino)(N-methyl-N-phenylcarbamoyl)sulfide Data collection

**Table:**

Bruker SMART APEXII diffractometer	2961 reflections with *I* > 2σ(*I*)
φ and ω scans	*R*_int_ = 0.022
Absorption correction: multi-scan (SADABS; Bruker, 2002)	θ_max_ = 27.4°, θ_min_ = 1.8°
*T*_min_ = 0.687, *T*_max_ = 0.746	*h* = −11→11
8061 measured reflections	*k* = −8→8
3145 independent reflections	*l* = −14→14

### (I) (N-Methyl-N-phenylamino)(N-methyl-N-phenylcarbamoyl)sulfide Refinement

**Table:**

Refinement on *F*^2^	Hydrogen site location: inferred from neighbouring sites
Least-squares matrix: full	H-atom parameters constrained
*R*[*F*^2^ > 2σ(*F*^2^)] = 0.027	*w* = 1/[σ^2^(*F*_o_^2^) + (0.0297*P*)^2^ + 0.1164*P*] where *P* = (*F*_o_^2^ + 2*F*_c_^2^)/3
*wR*(*F*^2^) = 0.067	(Δ/σ)_max_ < 0.001
*S* = 1.05	Δρ_max_ = 0.19 e Å^−^^3^
3145 reflections	Δρ_min_ = −0.15 e Å^−^^3^
174 parameters	Absolute structure: Flack *x* determined using 1285 quotients [(*I*^+^)-(*I*^-^)]/[(*I*^+^)+(*I*^-^)] (Parsons *et al.*, 2013)
1 restraint	Absolute structure parameter: 0.05 (3)

### (I) (N-Methyl-N-phenylamino)(N-methyl-N-phenylcarbamoyl)sulfide Special details

**Table:**

Geometry. All e.s.d.'s are estimated using the full covariance matrix. The cell e.s.d.'s are taken into account individually in the estimation of e.s.d.'s in distances, angles and torsion angles; correlations between e.s.d.'s in cell parameters are only used when they are defined by crystal symmetry. An approximate (isotropic) treatment of cell e.s.d.'s is used for estimating e.s.d.'s involving l.s. planes.

### (I) (N-Methyl-N-phenylamino)(N-methyl-N-phenylcarbamoyl)sulfide Fractional atomic coordinates and isotropic or equivalent isotropic displacement parameters (Å^2^)

**Table:**

	*x*	*y*	*z*	*U*_iso_*/*U*_eq_	
S1	0.32124 (5)	0.41906 (8)	0.67886 (4)	0.02848 (13)	
O1	0.41605 (17)	0.0518 (2)	0.71103 (13)	0.0314 (3)	
N1	0.2797 (2)	0.1212 (3)	0.52292 (15)	0.0265 (4)	
N2	0.39549 (17)	0.4094 (3)	0.82703 (13)	0.0264 (3)	
C1	0.2020 (2)	0.2644 (3)	0.43921 (17)	0.0248 (4)	
C2	0.2834 (2)	0.3936 (4)	0.38553 (17)	0.0289 (5)	
H2A	0.3910	0.3938	0.4080	0.035*	
C3	0.2080 (3)	0.5227 (3)	0.2990 (2)	0.0352 (5)	
H3A	0.2637	0.6114	0.2619	0.042*	
C4	0.0517 (3)	0.5218 (4)	0.2669 (2)	0.0383 (6)	
H4A	−0.0001	0.6109	0.2079	0.046*	
C5	−0.0302 (2)	0.3922 (4)	0.31988 (19)	0.0391 (6)	
H5A	−0.1378	0.3926	0.2972	0.047*	
C6	0.0446 (2)	0.2620 (4)	0.40601 (19)	0.0317 (5)	
H6A	−0.0112	0.1719	0.4420	0.038*	
C7	0.2839 (2)	−0.0795 (4)	0.47987 (18)	0.0317 (4)	
H7A	0.3168	−0.1675	0.5483	0.048*	
H7B	0.3553	−0.0877	0.4276	0.048*	
H7C	0.1826	−0.1176	0.4348	0.048*	
C8	0.3469 (2)	0.1659 (3)	0.63758 (18)	0.0247 (4)	
C9	0.3038 (3)	0.3084 (4)	0.8984 (2)	0.0330 (5)	
H9A	0.3380	0.3467	0.9827	0.050*	
H9B	0.3151	0.1668	0.8911	0.050*	
H9C	0.1970	0.3443	0.8691	0.050*	
C10	0.5557 (2)	0.4074 (4)	0.86869 (15)	0.0248 (4)	
C11	0.6441 (3)	0.5337 (4)	0.81829 (18)	0.0317 (5)	
H11A	0.5971	0.6177	0.7545	0.038*	
C12	0.8000 (3)	0.5379 (4)	0.8604 (2)	0.0364 (5)	
H12A	0.8592	0.6238	0.8248	0.044*	
C13	0.8703 (2)	0.4179 (5)	0.95412 (18)	0.0363 (5)	
H13A	0.9774	0.4200	0.9825	0.044*	
C14	0.7824 (3)	0.2954 (4)	1.0056 (2)	0.0357 (5)	
H14A	0.8297	0.2139	1.0706	0.043*	
C15	0.6264 (3)	0.2891 (3)	0.9641 (2)	0.0306 (5)	
H15A	0.5676	0.2041	1.0008	0.037*	

### (I) (N-Methyl-N-phenylamino)(N-methyl-N-phenylcarbamoyl)sulfide Atomic displacement parameters (Å^2^)

**Table:**

	*U*^11^	*U*^22^	*U*^33^	*U*^12^	*U*^13^	*U*^23^
S1	0.0321 (2)	0.0234 (2)	0.0263 (2)	0.0027 (2)	−0.00070 (17)	−0.0012 (2)
O1	0.0339 (8)	0.0284 (8)	0.0284 (8)	0.0061 (6)	0.0002 (6)	0.0003 (7)
N1	0.0291 (9)	0.0245 (9)	0.0240 (8)	−0.0005 (7)	0.0019 (7)	−0.0019 (7)
N2	0.0278 (8)	0.0279 (8)	0.0232 (7)	−0.0030 (9)	0.0053 (6)	−0.0007 (9)
C1	0.0266 (10)	0.0259 (10)	0.0207 (10)	0.0016 (8)	0.0031 (8)	−0.0037 (8)
C2	0.0283 (10)	0.0291 (13)	0.0273 (9)	−0.0012 (9)	0.0021 (8)	−0.0029 (9)
C3	0.0461 (13)	0.0285 (12)	0.0294 (11)	−0.0029 (10)	0.0054 (10)	−0.0002 (9)
C4	0.0475 (14)	0.0365 (13)	0.0268 (11)	0.0149 (11)	−0.0002 (10)	0.0002 (10)
C5	0.0275 (10)	0.0534 (17)	0.0340 (11)	0.0107 (12)	0.0024 (8)	−0.0059 (12)
C6	0.0253 (10)	0.0419 (13)	0.0285 (11)	0.0011 (9)	0.0075 (8)	−0.0026 (9)
C7	0.0364 (10)	0.0276 (9)	0.0309 (10)	0.0006 (12)	0.0073 (8)	−0.0040 (12)
C8	0.0222 (9)	0.0231 (10)	0.0287 (10)	−0.0003 (8)	0.0059 (8)	−0.0021 (8)
C9	0.0323 (11)	0.0330 (12)	0.0355 (12)	−0.0054 (10)	0.0115 (9)	−0.0031 (10)
C10	0.0292 (9)	0.0252 (9)	0.0197 (8)	−0.0028 (10)	0.0049 (7)	−0.0053 (9)
C11	0.0363 (11)	0.0364 (12)	0.0218 (10)	−0.0061 (10)	0.0055 (9)	0.0016 (9)
C12	0.0351 (12)	0.0457 (14)	0.0302 (11)	−0.0135 (11)	0.0115 (10)	−0.0034 (10)
C13	0.0286 (10)	0.0442 (12)	0.0348 (10)	−0.0034 (13)	0.0045 (8)	−0.0094 (14)
C14	0.0363 (12)	0.0343 (11)	0.0322 (12)	0.0010 (10)	−0.0007 (9)	0.0006 (10)
C15	0.0345 (11)	0.0261 (10)	0.0307 (11)	−0.0027 (10)	0.0069 (9)	0.0017 (9)

### (I) (N-Methyl-N-phenylamino)(N-methyl-N-phenylcarbamoyl)sulfide Geometric parameters (Å, º)

**Table:**

S1—N2	1.6784 (15)	C6—H6A	0.9500
S1—C8	1.824 (2)	C7—H7A	0.9800
O1—C8	1.212 (3)	C7—H7B	0.9800
N1—C8	1.351 (3)	C7—H7C	0.9800
N1—C1	1.437 (3)	C9—H9A	0.9800
N1—C7	1.462 (3)	C9—H9B	0.9800
N2—C10	1.421 (2)	C9—H9C	0.9800
N2—C9	1.467 (3)	C10—C11	1.392 (3)
C1—C2	1.383 (3)	C10—C15	1.393 (3)
C1—C6	1.390 (3)	C11—C12	1.385 (3)
C2—C3	1.385 (3)	C11—H11A	0.9500
C2—H2A	0.9500	C12—C13	1.385 (4)
C3—C4	1.380 (3)	C12—H12A	0.9500
C3—H3A	0.9500	C13—C14	1.379 (4)
C4—C5	1.384 (4)	C13—H13A	0.9500
C4—H4A	0.9500	C14—C15	1.385 (3)
C5—C6	1.386 (3)	C14—H14A	0.9500
C5—H5A	0.9500	C15—H15A	0.9500
			
N2—S1—C8	100.36 (10)	H7A—C7—H7C	109.5
C8—N1—C1	122.48 (18)	H7B—C7—H7C	109.5
C8—N1—C7	120.04 (17)	O1—C8—N1	125.12 (19)
C1—N1—C7	117.48 (16)	O1—C8—S1	120.52 (16)
C10—N2—C9	118.37 (17)	N1—C8—S1	114.34 (15)
C10—N2—S1	118.74 (12)	N2—C9—H9A	109.5
C9—N2—S1	115.90 (14)	N2—C9—H9B	109.5
C2—C1—C6	120.34 (19)	H9A—C9—H9B	109.5
C2—C1—N1	120.23 (18)	N2—C9—H9C	109.5
C6—C1—N1	119.25 (19)	H9A—C9—H9C	109.5
C1—C2—C3	120.05 (19)	H9B—C9—H9C	109.5
C1—C2—H2A	120.0	C11—C10—C15	118.84 (18)
C3—C2—H2A	120.0	C11—C10—N2	119.8 (2)
C4—C3—C2	119.7 (2)	C15—C10—N2	121.28 (19)
C4—C3—H3A	120.2	C12—C11—C10	120.5 (2)
C2—C3—H3A	120.2	C12—C11—H11A	119.8
C3—C4—C5	120.5 (2)	C10—C11—H11A	119.8
C3—C4—H4A	119.7	C11—C12—C13	120.6 (2)
C5—C4—H4A	119.7	C11—C12—H12A	119.7
C4—C5—C6	120.1 (2)	C13—C12—H12A	119.7
C4—C5—H5A	120.0	C14—C13—C12	118.91 (19)
C6—C5—H5A	120.0	C14—C13—H13A	120.5
C5—C6—C1	119.4 (2)	C12—C13—H13A	120.5
C5—C6—H6A	120.3	C13—C14—C15	121.1 (2)
C1—C6—H6A	120.3	C13—C14—H14A	119.4
N1—C7—H7A	109.5	C15—C14—H14A	119.4
N1—C7—H7B	109.5	C14—C15—C10	120.0 (2)
H7A—C7—H7B	109.5	C14—C15—H15A	120.0
N1—C7—H7C	109.5	C10—C15—H15A	120.0
			
C8—S1—N2—C10	77.3 (2)	C1—N1—C8—S1	3.3 (2)
C8—S1—N2—C9	−73.05 (18)	C7—N1—C8—S1	−176.52 (14)
C8—N1—C1—C2	78.2 (3)	N2—S1—C8—O1	−6.02 (19)
C7—N1—C1—C2	−101.9 (2)	N2—S1—C8—N1	172.19 (14)
C8—N1—C1—C6	−106.6 (2)	C9—N2—C10—C11	−166.6 (2)
C7—N1—C1—C6	73.2 (2)	S1—N2—C10—C11	43.8 (3)
C6—C1—C2—C3	0.6 (3)	C9—N2—C10—C15	9.7 (3)
N1—C1—C2—C3	175.69 (19)	S1—N2—C10—C15	−139.90 (19)
C1—C2—C3—C4	0.1 (3)	C15—C10—C11—C12	1.6 (3)
C2—C3—C4—C5	−0.4 (3)	N2—C10—C11—C12	178.0 (2)
C3—C4—C5—C6	0.0 (4)	C10—C11—C12—C13	−0.6 (4)
C4—C5—C6—C1	0.6 (3)	C11—C12—C13—C14	−0.6 (4)
C2—C1—C6—C5	−0.9 (3)	C12—C13—C14—C15	0.8 (4)
N1—C1—C6—C5	−176.1 (2)	C13—C14—C15—C10	0.2 (4)
C1—N1—C8—O1	−178.6 (2)	C11—C10—C15—C14	−1.4 (3)
C7—N1—C8—O1	1.6 (3)	N2—C10—C15—C14	−177.7 (2)

### (II) (N-Methyl-N-phenylamino)(N-methyl-N-phenylcarbamoyl)disulfane Crystal data

**Table:**

C_15_H_16_N_2_OS_2_	*F*(000) = 640
*M**_r_* = 304.42	*D*_x_ = 1.375 Mg m^−^^3^
Monoclinic, *P*2_1_/*c*	Mo *K*α radiation, λ = 0.71073 Å
*a* = 16.0414 (17) Å	Cell parameters from 2950 reflections
*b* = 5.5023 (6) Å	θ = 3.1–27.5°
*c* = 17.2986 (19) Å	µ = 0.36 mm^−^^1^
β = 105.564 (1)°	*T* = 123 K
*V* = 1470.9 (3) Å^3^	Block, colorless
*Z* = 4	0.41 × 0.18 × 0.12 mm

### (II) (N-Methyl-N-phenylamino)(N-methyl-N-phenylcarbamoyl)disulfane Data collection

**Table:**

Bruker APEXII CCD diffractometer	3033 reflections with *I* > 2σ(*I*)
Radiation source: sealed tube	*R*_int_ = 0.024
φ and ω scans	θ_max_ = 27.5°, θ_min_ = 1.3°
Absorption correction: multi-scan (SADABS; Bruker, 2002)	*h* = −20→20
*T*_min_ = 0.699, *T*_max_ = 0.746	*k* = −7→7
16044 measured reflections	*l* = −22→22
3355 independent reflections	

### (II) (N-Methyl-N-phenylamino)(N-methyl-N-phenylcarbamoyl)disulfane Refinement

**Table:**

Refinement on *F*^2^	0 restraints
Least-squares matrix: full	Hydrogen site location: inferred from neighbouring sites
*R*[*F*^2^ > 2σ(*F*^2^)] = 0.028	H-atom parameters constrained
*wR*(*F*^2^) = 0.070	*w* = 1/[σ^2^(*F*_o_^2^) + (0.0301*P*)^2^ + 0.7728*P*] where *P* = (*F*_o_^2^ + 2*F*_c_^2^)/3
*S* = 1.06	(Δ/σ)_max_ < 0.001
3355 reflections	Δρ_max_ = 0.32 e Å^−^^3^
183 parameters	Δρ_min_ = −0.22 e Å^−^^3^

### (II) (N-Methyl-N-phenylamino)(N-methyl-N-phenylcarbamoyl)disulfane Special details

**Table:**

Geometry. All e.s.d.'s are estimated using the full covariance matrix. The cell e.s.d.'s are taken into account individually in the estimation of e.s.d.'s in distances, angles and torsion angles; correlations between e.s.d.'s in cell parameters are only used when they are defined by crystal symmetry. An approximate (isotropic) treatment of cell e.s.d.'s is used for estimating e.s.d.'s involving l.s. planes.

### (II) (N-Methyl-N-phenylamino)(N-methyl-N-phenylcarbamoyl)disulfane Fractional atomic coordinates and isotropic or equivalent isotropic displacement parameters (Å^2^)

**Table:**

	*x*	*y*	*z*	*U*_iso_*/*U*_eq_	
S1	0.24264 (2)	0.27366 (6)	0.12829 (2)	0.01720 (9)	
S2	0.27516 (2)	0.52421 (6)	0.05260 (2)	0.01821 (9)	
O1	0.15803 (6)	0.07736 (18)	−0.01294 (5)	0.0215 (2)	
N1	0.12052 (7)	−0.0673 (2)	0.09646 (6)	0.0173 (2)	
N2	0.36329 (7)	0.4255 (2)	0.02877 (6)	0.0184 (2)	
C1	0.12310 (8)	−0.0405 (2)	0.18005 (7)	0.0160 (2)	
C2	0.16345 (8)	−0.2156 (2)	0.23508 (8)	0.0199 (3)	
H2A	0.1907	−0.3512	0.2182	0.024*	
C3	0.16379 (9)	−0.1914 (3)	0.31517 (8)	0.0232 (3)	
H3A	0.1907	−0.3119	0.3530	0.028*	
C4	0.12493 (9)	0.0083 (3)	0.33984 (8)	0.0227 (3)	
H4A	0.1266	0.0268	0.3948	0.027*	
C5	0.08363 (9)	0.1808 (3)	0.28448 (8)	0.0239 (3)	
H5A	0.0565	0.3166	0.3015	0.029*	
C6	0.08171 (8)	0.1558 (2)	0.20413 (8)	0.0209 (3)	
H6A	0.0523	0.2721	0.1659	0.025*	
C7	0.06129 (8)	−0.2499 (2)	0.05015 (8)	0.0204 (3)	
H7A	0.0626	−0.2431	−0.0061	0.031*	
H7B	0.0024	−0.2168	0.0537	0.031*	
H7C	0.0792	−0.4118	0.0719	0.031*	
C8	0.16585 (8)	0.0771 (2)	0.05874 (7)	0.0163 (2)	
C9	0.34991 (9)	0.2336 (3)	−0.03205 (8)	0.0233 (3)	
H9A	0.3968	0.2384	−0.0585	0.035*	
H9B	0.2943	0.2589	−0.0720	0.035*	
H9C	0.3497	0.0750	−0.0063	0.035*	
C10	0.44464 (8)	0.4352 (2)	0.08806 (7)	0.0168 (2)	
C11	0.50700 (9)	0.2568 (2)	0.09139 (8)	0.0219 (3)	
H11A	0.4951	0.1250	0.0546	0.026*	
C12	0.58679 (9)	0.2706 (3)	0.14843 (9)	0.0256 (3)	
H12A	0.6291	0.1485	0.1500	0.031*	
C13	0.60500 (9)	0.4600 (3)	0.20266 (8)	0.0242 (3)	
H13A	0.6594	0.4686	0.2416	0.029*	
C14	0.54283 (9)	0.6379 (3)	0.19970 (8)	0.0228 (3)	
H14A	0.5548	0.7683	0.2371	0.027*	
C15	0.46357 (9)	0.6273 (2)	0.14279 (8)	0.0202 (3)	
H15A	0.4219	0.7512	0.1410	0.024*	

### (II) (N-Methyl-N-phenylamino)(N-methyl-N-phenylcarbamoyl)disulfane Atomic displacement parameters (Å^2^)

**Table:**

	*U*^11^	*U*^22^	*U*^33^	*U*^12^	*U*^13^	*U*^23^
S1	0.01726 (16)	0.01986 (16)	0.01529 (15)	−0.00238 (11)	0.00576 (12)	−0.00051 (11)
S2	0.01769 (16)	0.01601 (16)	0.02234 (16)	0.00171 (11)	0.00784 (12)	0.00252 (12)
O1	0.0234 (5)	0.0262 (5)	0.0148 (4)	−0.0021 (4)	0.0049 (4)	0.0012 (4)
N1	0.0179 (5)	0.0187 (5)	0.0158 (5)	−0.0033 (4)	0.0055 (4)	−0.0012 (4)
N2	0.0170 (5)	0.0226 (6)	0.0175 (5)	−0.0012 (4)	0.0079 (4)	−0.0021 (4)
C1	0.0150 (6)	0.0183 (6)	0.0159 (6)	−0.0029 (5)	0.0063 (4)	−0.0013 (5)
C2	0.0203 (6)	0.0192 (6)	0.0208 (6)	0.0021 (5)	0.0066 (5)	−0.0011 (5)
C3	0.0234 (7)	0.0254 (7)	0.0192 (6)	0.0008 (5)	0.0030 (5)	0.0033 (5)
C4	0.0231 (7)	0.0293 (7)	0.0177 (6)	−0.0064 (5)	0.0088 (5)	−0.0050 (5)
C5	0.0269 (7)	0.0203 (7)	0.0299 (7)	−0.0006 (5)	0.0168 (6)	−0.0042 (5)
C6	0.0211 (6)	0.0193 (6)	0.0251 (7)	0.0025 (5)	0.0110 (5)	0.0037 (5)
C7	0.0197 (6)	0.0204 (6)	0.0198 (6)	−0.0034 (5)	0.0031 (5)	−0.0009 (5)
C8	0.0144 (6)	0.0161 (6)	0.0181 (6)	0.0020 (5)	0.0039 (5)	0.0013 (5)
C9	0.0211 (6)	0.0322 (8)	0.0173 (6)	−0.0031 (5)	0.0064 (5)	−0.0068 (5)
C10	0.0177 (6)	0.0185 (6)	0.0165 (6)	−0.0035 (5)	0.0087 (5)	0.0004 (5)
C11	0.0235 (7)	0.0204 (7)	0.0225 (6)	−0.0008 (5)	0.0074 (5)	−0.0064 (5)
C12	0.0212 (7)	0.0255 (7)	0.0295 (7)	0.0026 (5)	0.0056 (6)	−0.0049 (6)
C13	0.0198 (6)	0.0292 (7)	0.0228 (7)	−0.0040 (5)	0.0042 (5)	−0.0027 (6)
C14	0.0264 (7)	0.0220 (7)	0.0218 (6)	−0.0060 (5)	0.0098 (5)	−0.0069 (5)
C15	0.0226 (6)	0.0177 (6)	0.0230 (6)	−0.0014 (5)	0.0106 (5)	−0.0023 (5)

### (II) (N-Methyl-N-phenylamino)(N-methyl-N-phenylcarbamoyl)disulfane Geometric parameters (Å, º)

**Table:**

S1—C8	1.8273 (13)	C6—H6A	0.9500
S1—S2	2.0625 (5)	C7—H7A	0.9800
S2—N2	1.6660 (11)	C7—H7B	0.9800
O1—C8	1.2123 (15)	C7—H7C	0.9800
N1—C8	1.3569 (16)	C9—H9A	0.9800
N1—C1	1.4429 (15)	C9—H9B	0.9800
N1—C7	1.4646 (16)	C9—H9C	0.9800
N2—C10	1.4281 (16)	C10—C11	1.3917 (18)
N2—C9	1.4656 (16)	C10—C15	1.3967 (18)
C1—C2	1.3865 (18)	C11—C12	1.3929 (19)
C1—C6	1.3888 (18)	C11—H11A	0.9500
C2—C3	1.3905 (18)	C12—C13	1.3800 (19)
C2—H2A	0.9500	C12—H12A	0.9500
C3—C4	1.386 (2)	C13—C14	1.388 (2)
C3—H3A	0.9500	C13—H13A	0.9500
C4—C5	1.384 (2)	C14—C15	1.3847 (19)
C4—H4A	0.9500	C14—H14A	0.9500
C5—C6	1.3888 (18)	C15—H15A	0.9500
C5—H5A	0.9500		
			
C8—S1—S2	102.60 (4)	H7A—C7—H7C	109.5
N2—S2—S1	108.37 (4)	H7B—C7—H7C	109.5
C8—N1—C1	123.17 (10)	O1—C8—N1	124.80 (12)
C8—N1—C7	119.43 (10)	O1—C8—S1	122.64 (10)
C1—N1—C7	117.28 (10)	N1—C8—S1	112.55 (9)
C10—N2—C9	118.17 (11)	N2—C9—H9A	109.5
C10—N2—S2	118.86 (8)	N2—C9—H9B	109.5
C9—N2—S2	116.23 (8)	H9A—C9—H9B	109.5
C2—C1—C6	120.47 (11)	N2—C9—H9C	109.5
C2—C1—N1	119.97 (11)	H9A—C9—H9C	109.5
C6—C1—N1	119.51 (11)	H9B—C9—H9C	109.5
C1—C2—C3	119.60 (12)	C11—C10—C15	118.84 (12)
C1—C2—H2A	120.2	C11—C10—N2	120.77 (11)
C3—C2—H2A	120.2	C15—C10—N2	120.38 (12)
C4—C3—C2	120.05 (13)	C10—C11—C12	120.30 (12)
C4—C3—H3A	120.0	C10—C11—H11A	119.8
C2—C3—H3A	120.0	C12—C11—H11A	119.8
C5—C4—C3	120.12 (12)	C13—C12—C11	120.62 (13)
C5—C4—H4A	119.9	C13—C12—H12A	119.7
C3—C4—H4A	119.9	C11—C12—H12A	119.7
C4—C5—C6	120.16 (12)	C12—C13—C14	119.24 (13)
C4—C5—H5A	119.9	C12—C13—H13A	120.4
C6—C5—H5A	119.9	C14—C13—H13A	120.4
C1—C6—C5	119.55 (12)	C15—C14—C13	120.65 (12)
C1—C6—H6A	120.2	C15—C14—H14A	119.7
C5—C6—H6A	120.2	C13—C14—H14A	119.7
N1—C7—H7A	109.5	C14—C15—C10	120.34 (12)
N1—C7—H7B	109.5	C14—C15—H15A	119.8
H7A—C7—H7B	109.5	C10—C15—H15A	119.8
N1—C7—H7C	109.5		
			
S1—S2—N2—C10	−72.86 (10)	C7—N1—C8—S1	−175.04 (9)
S1—S2—N2—C9	77.90 (9)	C8—S1—S2—N2	−92.62 (6)
C8—N1—C1—C2	−110.35 (14)	S2—S1—C8—O1	15.13 (12)
C7—N1—C1—C2	73.77 (15)	S2—S1—C8—N1	−165.53 (8)
C8—N1—C1—C6	72.46 (16)	C9—N2—C10—C11	−4.59 (17)
C7—N1—C1—C6	−103.41 (14)	S2—N2—C10—C11	145.60 (11)
C6—C1—C2—C3	−1.25 (19)	C9—N2—C10—C15	174.12 (11)
N1—C1—C2—C3	−178.41 (12)	S2—N2—C10—C15	−35.69 (15)
C1—C2—C3—C4	−0.8 (2)	C15—C10—C11—C12	−0.02 (19)
C2—C3—C4—C5	1.8 (2)	N2—C10—C11—C12	178.70 (12)
C3—C4—C5—C6	−0.6 (2)	C10—C11—C12—C13	0.4 (2)
C2—C1—C6—C5	2.36 (19)	C11—C12—C13—C14	−0.2 (2)
N1—C1—C6—C5	179.54 (12)	C12—C13—C14—C15	−0.4 (2)
C4—C5—C6—C1	−1.4 (2)	C13—C14—C15—C10	0.8 (2)
C1—N1—C8—O1	−171.52 (12)	C11—C10—C15—C14	−0.56 (19)
C7—N1—C8—O1	4.27 (19)	N2—C10—C15—C14	−179.29 (11)
C1—N1—C8—S1	9.16 (15)		

### (II) (N-Methyl-N-phenylamino)(N-methyl-N-phenylcarbamoyl)disulfane Hydrogen-bond geometry (Å, º)

**Table:**

*D*—H···*A*	*D*—H	H···*A*	*D*···*A*	*D*—H···*A*
C2—H2*A*···S1^i^	0.95	2.84	3.766 (1)	165
C7—H7*B*···O1^ii^	0.98	2.60	3.532 (2)	160

Symmetry codes: (i) *x*, *y*−1, *z*; (ii) −*x*, −*y*, −*z*.
